# Gastric Perforation Due to Fungal Infection in a Diabetic Patient: A Case Report and Review of the Literature

**DOI:** 10.7759/cureus.96868

**Published:** 2025-11-14

**Authors:** Omar S Alani, Musa'ab N Omair, Mohamed Dabbagh, Nafe Alhariri, Abdullah Alani

**Affiliations:** 1 General Surgery, Al-Qassimi Hospital, Sharjah, ARE; 2 General Surgery, Mohammed Bin Rashed University, Dubai, ARE; 3 Basic Sciences, Ajman University, Ajman, ARE

**Keywords:** candida albicans, case report, fungal peritonitis, gastric perforation, intensive care

## Abstract

Gastric perforation is a life-threatening surgical emergency most commonly caused by peptic ulcer disease. Fungal involvement, particularly by Candida albicans, is exceedingly rare but associated with poorer outcomes than bacterial peritonitis. We report a case of a 52-year-old man with type 2 diabetes mellitus who presented with acute abdominal pain and hemodynamic instability. Emergency laparotomy revealed a 5 mm perforation on the anterior gastric wall, which was repaired primarily. Postoperatively, the patient developed persistent sepsis, acute kidney injury, and hypoalbuminemia. Two weeks later, imaging showed focal dehiscence at the repair site. Intra-abdominal cultures grew *Candida albicans *and *Pseudomonas aeruginosa*, while blood cultures confirmed candidemia. Periodic acid-Schiff (PAS) staining demonstrated fungus-hyphae consistent with invasive gastric candidiasis. Despite antifungal therapy, the patient’s condition deteriorated, necessitating re-exploration, where a friable gastric wall precluded re-repair. Candida albicans-associated gastric perforation is exceptionally uncommon, with less than 15 cases reported in the literature. Diabetes, malnutrition, and prolonged antibiotic exposure are key risk factors. Early recognition of fungal involvement, timely antifungal therapy, and tailored surgical management are critical to improving outcomes. This case highlights the importance of considering fungal etiologies in refractory intra-abdominal sepsis.

## Introduction

Gastric perforation is a surgical emergency most commonly associated with peptic ulcer disease and is linked to significant morbidity and mortality. While bacterial contamination has long been recognized as the main contributor to peritonitis in this setting, fungal involvement is increasingly reported and is associated with worse outcomes [1]. Candida species are part of the gut flora, but their proliferation in the stomach is usually suppressed by the acidic gastric environment [2]. Alterations in gastric pH caused by proton pump inhibitor (PPI) therapy, surgery, or critical illness may disrupt this barrier, allowing overgrowth and potential invasion [3]. Nosocomial factors such as broad-spectrum antibiotic use, mechanical ventilation, and intensive care admission further increase the risk of invasive candidiasis [2]. Several clinical studies have confirmed that fungal isolation in perforated peptic ulcer (PPU) peritonitis is not incidental. For example, Lee et al. reported Candida species in 37% of patients, with mortality significantly higher than in culture-negative patients [1]. A prospective study involving 84 patients with gastrointestinal perforation and peritonitis found that those with positive fungal cultures had poorer outcomes [4]. These findings were later reinforced by Jindal et al. [5], which demonstrated higher morbidity among patients with Candida-positive cultures.

This case report aims to describe a rare presentation of Candida albicans-associated gastric perforation, complicated by postoperative fungal peritonitis and Candidemia. The patient’s course was further complicated by diabetic ketoacidosis, sepsis, intubation and ICU admission. This combination of clinical complications is rarely reported, making this case noteworthy. By presenting this report, we aim to highlight the diagnostic challenges, therapeutic considerations, and the need for heightened suspicion of fungal involvement in critically ill patients with gastric ulcer perforation.

## Case presentation

A 52-year-old male of North Asian descent with a history of type 2 diabetes mellitus presented to the emergency department of a tertiary hospital in August 2025 (day 0). The patient complained of abdominal pain that started a few hours prior. The pain started suddenly, was severe and constant, and he rated it 10 out of 10. He also reported nausea without vomiting, and constipation that had begun prior to the onset of pain. Before presenting to our hospital, the patient had been admitted to another hospital and received analgesia, but the pain returned and worsened over time. He denied having a history of fever, weight loss, fatigue, irritability, episodes of pain, abdominal surgeries, or chronic ulcers. He has been a smoker for many years, but denied long-term use of NSAIDs or PPIs, alcohol use, or allergies to any medications. His diabetes is controlled on 800 mg of metformin daily. The patient was unable to lie down due to the severity of pain, and he was examined in the semi-sitting position. On arrival, he had a temperature of 37°C, HR 108, RR 16, BP 133/86, SpO_2_ 98%. On the initial physical exam, the patient was conscious, alert, and oriented to time and place. On general inspection, there was no pallor, icterus, cyanosis, or digital clubbing. In addition, no bilateral edema or organomegaly was noted, and the jugular venous pressure (JVP) was within normal range. On chest examination, auscultation of the heart revealed normal S1 and S2 with no added sounds. The lungs were clear, with bilateral air entry, no added sounds, and full chest expansion. On abdominal examination, there was generalized tenderness with rigidity and guarding. Blood samples were collected in the emergency department for laboratory testing, which revealed marked leukocytosis, with normal hemoglobin. Platelets were significantly reduced and renal function tests were consistent with acute kidney injury. Uric acid was elevated, and blood glucose was markedly raised. The coagulation profile showed prolonged prothrombin time (PT), partial thromboplastin time (PTT), and international normalized ratio (INR), indicating coagulopathy. Electrolytes demonstrated hypernatremia, in addition to elevation in inflammatory markers. These findings were consistent with severe sepsis, multi-organ dysfunction, and a compromised physiological reserve at presentation. A provisional diagnosis of acute abdomen was made.

**Figure 1:**
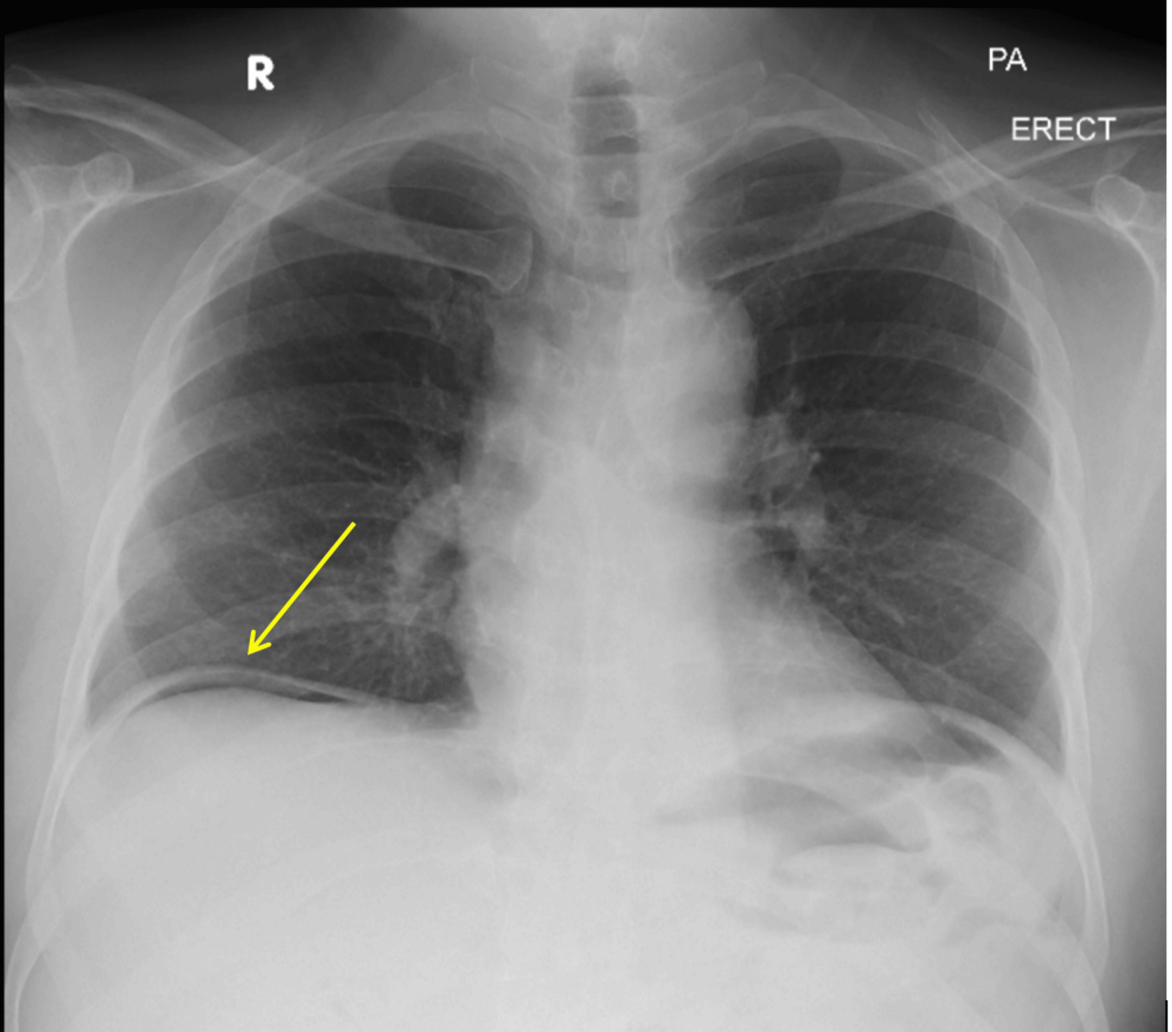
Upright Chest X-ray showing air under the diaphragm

In the emergency department, the patient was started on 500 mL of IV fluid over one hour, a one-time dose of 5 mg IV morphine and 75 mg IM diclofenac for analgesia. Blood was sent for ABO/Rh typing, serology testing for hepatitis B surface antigen, hepatitis C antibody, and HIV screening, all in preparation for emergency surgery. An initial upright X-ray of the abdomen revealed air under the diaphragm, as shown in Figure 1.

General surgery was consulted, and the decision was made to proceed with an emergency exploratory laparotomy. A rapid pre-operative anesthesia assessment classified the patient as ASA Class IIE. His last oral intake was approximately 8 hours prior to the procedure (NPO since 12 AM). Physical assessment for anesthesia revealed a short neck and a Mallampati Class IV. Jaw mobility and mouth opening were adequate, and C-spine mobility was normal. The patient's pre-procedure vital signs showed a temperature of 37°C, an unmonitored heart rate, RR 18, BP 140/80 mmHg, and an SpO_2_ of 100%. The ECG showed a normal sinus rhythm. The planned anesthetic technique was general anesthesia with a rapid sequence induction (RSI). After discussing the risks, the patient consented to the anesthesia plan. He was subsequently transferred to the operating room, where exploratory laparotomy was performed under general anesthesia with endotracheal intubation. A nasogastric tube (NGT) and a Foley catheter were inserted, and standard intraoperative monitoring was employed.

Intraoperatively, the abdomen was entered through an upper median incision. Findings included turbid fluid present throughout the abdomen, and a 5 mm perforation was identified on the anterior wall of the stomach at the body, with active bile leakage. The estimated blood loss was 30 mL. The perforation was closed and sealed securely with a few interrupted sutures, and an omental patch (Graham patch) was then used to reinforce the repair. No further leakage was observed. The abdominal cavity was thoroughly washed with a large volume of warm saline. Two tube drains were inserted: one drain placed in the left pelvic cavity and another in the right subhepatic space. The abdomen was then closed in layers and a biopsy from the edges of the perforation was sent for pathological analysis. The patient was extubated and sent to the critical care unit.

Postoperatively and in the first few hours, the patient was alert and oriented. He was mildly tachycardic but maintained normal blood pressure and oxygen saturation. Postoperative care focused on keeping him NPO and on IV fluids, monitoring his vital signs, and surgical drain output. A patient-controlled analgesia (PCA) pump with morphine was initiated for pain control. The NGT remained in place for gastric decompression, and the patient's diabetes management was converted to an insulin regimen postoperatively.

Twenty-four hours after the surgery, the patient’s condition deteriorated. He developed tachypnea, tachycardia, hypotension (HR 135 bpm, BP 110/70 mmHg), and was noted to be drowsy but arousable. Arterial blood gas revealed metabolic acidosis and ketonuria, consistent with metabolic decompensation. Laboratory studies revealed acute kidney injury and hyperglycemia with elevated inflammatory markers, indicating diabetic ketoacidosis. Despite initiation of diabetic ketoacidosis (DKA) protocol (IV fluids and insulin infusion), the patient’s urine output did not improve. He required escalating respiratory support with high-flow nasal cannula (60 L/min, FiO₂ 60%) but continued to desaturate. A norepinephrine infusion at 10 mcg/min was started for hemodynamic support, and broad-spectrum antibiotics were escalated to meropenem and teicoplanin. A right internal jugular central venous catheter and left radial arterial line were inserted. He was reintubated and placed on mechanical ventilation. The patient remained critically ill under close monitoring. Total parenteral nutrition (TPN) was initiated due to intolerance of enteral feeding. Over the following days, the patient’s condition was complicated by persistent sepsis and abdominal distention. Serial laboratory results demonstrated marked leukocytosis, with WBCs remaining persistently high, consistent with ongoing intra-abdominal sepsis. Hemoglobin declined significantly by day 4, necessitating multiple transfusions. Renal function transiently improved over the following days, with creatinine decreasing to ~90 µmol/L by day 4, but later worsened again in the context of ongoing sepsis, rising above 200 µmol/L. Serum albumin remained persistently low, reflecting his poor nutritional status.

The intra-abdominal fluid culture grew Candida albicans and Pseudomonas aeruginosa, in addition to the blood culture showing Candida species. Histopathological examination of the specimen from the edges of perforation revealed fungal elements consistent with Candida albicans. Budding yeast cells and pseudohyphae within necrotic gastric tissue were seen. The organisms were highlighted using periodic acid-Schiff (PAS) stain, confirming the presence of C. albicans. The clinical picture concluded from the findings was invasive gastric candidiasis resulting in stomach perforation, fungal peritonitis, and candidemia.

**Figure 2:**
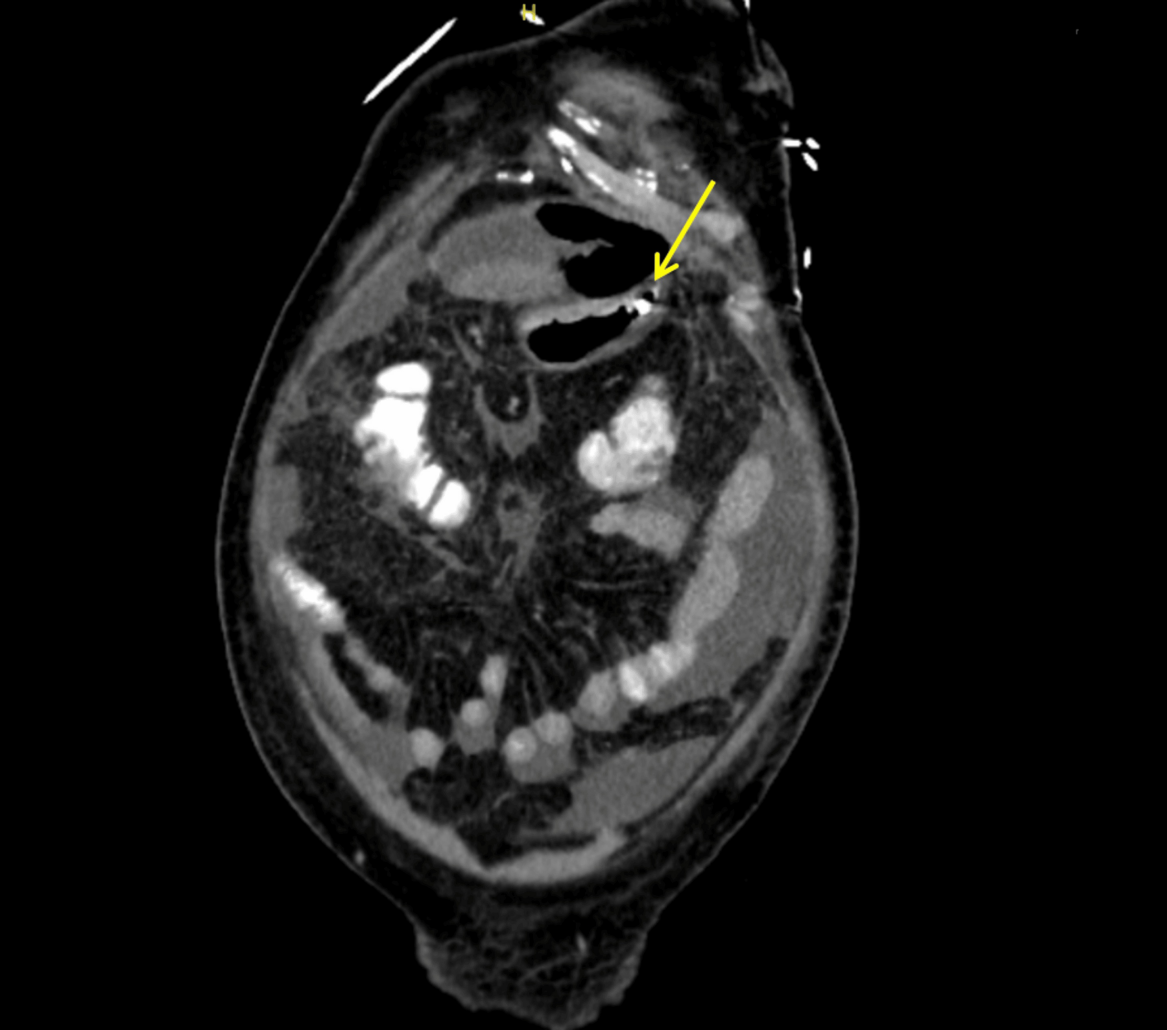
CT abdomen showing focal dehiscence of the anterior gastric wall with related air-fluid level

Imaging was performed in response to ongoing sepsis and clinical deterioration. A CT of the abdomen on day 2 demonstrated postoperative changes with mild free peritoneal fluid. A repeat CT on day 4 showed persistent intra-abdominal collections. A subsequent CT on day 13 demonstrated a leak at the repair site, in addition to suspected focal dehiscence of the anterior gastric wall with related air-fluid level, as shown in Figure 2. Despite broad-spectrum antibiotics and antifungal therapy, including amphotericin B, his condition worsened, prompting surgical re-exploration.

**Figure 3:**
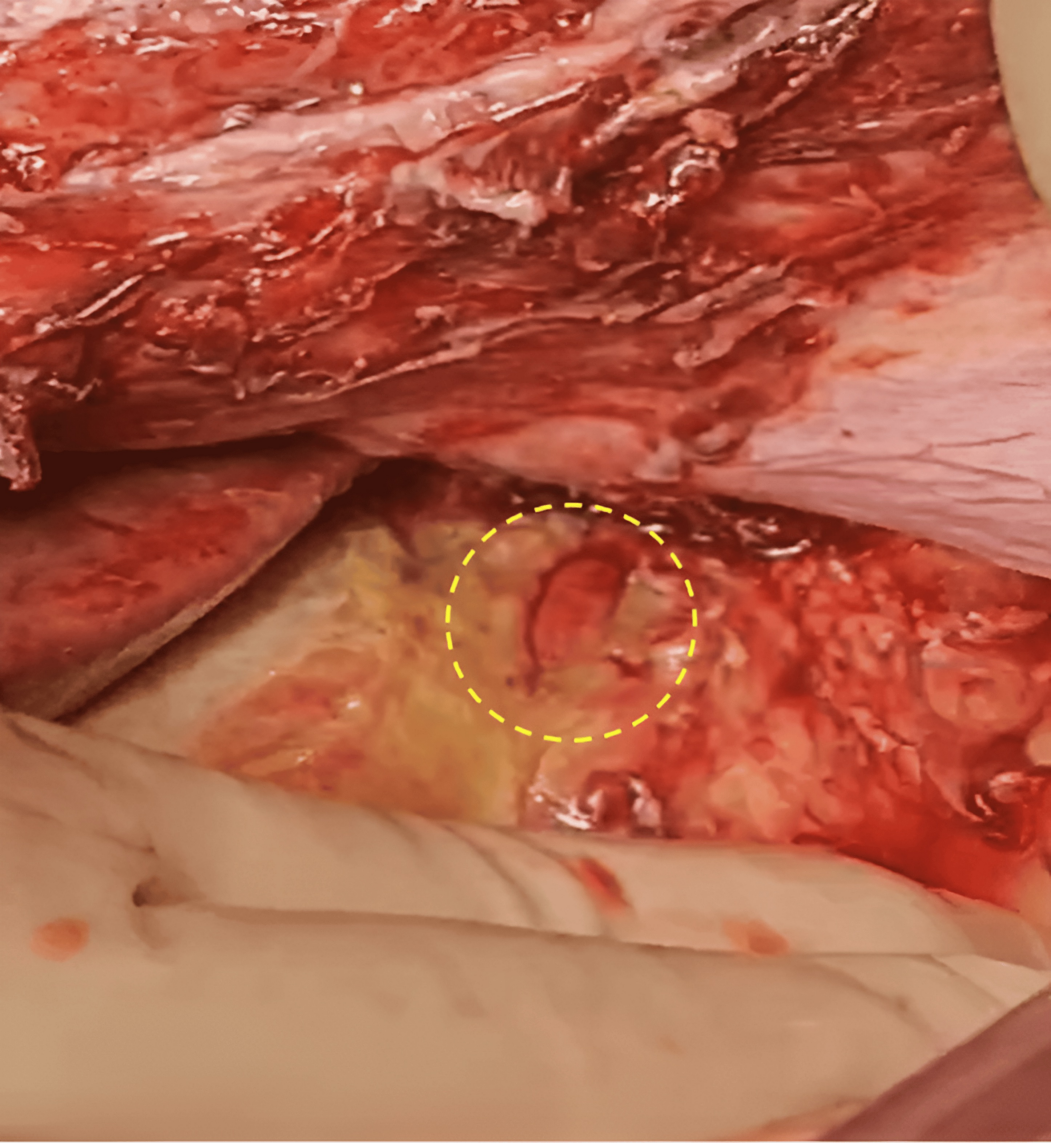
Intra-operative finding: re-perforation in the anterior stomach wall

Upon entering the abdomen, more than 2 L of purulent ascitic fluid were aspirated. Dense adhesions and a thickened, fibrosed omentum were encountered. Inspection revealed a leaking site at the previously repaired anterior gastric perforation, surrounded by friable, indurated gastric tissue (Figure 3).

Due to the friable gastric tissue, re‑suturing was not feasible. A gastrostomy tube was therefore inserted directly through the site of perforation and exteriorized through the abdominal wall for controlled drainage. Extensive peritoneal lavage was carried out using more than 4 L of warm saline, and two large drains were placed in the left upper quadrant (splenic bed) and pelvic cavity. A specimen of omentum was sent for histopathological analysis, which later showed acute suppurative inflammation with a chronic foreign body type reaction. Hemostasis was secured, and the abdomen was closed with tension sutures.

After the surgery, the patient was returned to the ICU, intubated, and sedated on SIMV ventilation. He was kept NPO, with the gastrostomy tube left on free drainage, and TPN was continued. Broad-spectrum antibiotics (including ceftazidime-avibactam) and antifungal therapy (amphotericin B) were administered, guided by microbiology results. Cultures from intra-abdominal fluid confirmed Candida albicans and Pseudomonas aeruginosa. The Pseudomonas isolate demonstrated resistance to meropenem but sensitivity to cefepime, ceftazidime, piperacillin-tazobactam, and ciprofloxacin. On day 14, the patient remained ventilated and sedated, with drains in situ and the gastrostomy tube draining. A methylene blue test was performed to assess for ongoing leakage. On day 15, TPN was increased to 2000 mL, and sedation was maintained with midazolam and fentanyl. On day 16, the methylene blue study was performed and showed no evidence of new leakage. Laboratory results in this period showed persistent leukocytosis, worsening anemia, and renal dysfunction with serum albumin remaining low at around 2.0 g/dL. A summary of values for the hematological and biochemical tests is shown in Table 1.

**Table 1:** Summary of Biochemical and Hematological Investigations (H): High, (L): Low, — : Not Available / Not Measured

Parameter	On Admission	Day 4	Day 14–18 (Post-2nd Surgery)	Reference Range
WBC (×10⁹/L)	19 (H)	12–15 (H)	13–15 (H)	4.0–10.0
Hemoglobin (g/dL)	15.2	8.5 (L)	6.2–8.8 (L)	13.5–17.5 (M)
Platelets (×10⁹/L)	85 (L)	—	—	150–400
Creatinine (µmol/L)	248 (H)	~90	>200 (H)	60–110
eGFR (mL/min/1.73m²)	26 (L)	—	—	>90
Uric Acid (µmol/L)	461 (H)	—	—	140–360
Blood Glucose (mmol/L)	17.2 (H)	—	—	3.9–7.8
PT (s)	18.4 (H)	—	—	11–14
PTT (s)	50.05 (H)	—	—	25–35
INR	1.62 (H)	—	—	0.8–1.2
Sodium (mmol/L)	152 (H)	—	—	135–145
Potassium (mmol/L)	4.1	—	—	3.5–5.0
Chloride (mmol/L)	117 (H)	—	—	98–107
Procalcitonin (ng/mL)	15.25 (H)	—	—	<0.05
CRP (mg/L)	246.7 (H)	—	—	<5
Albumin (g/dL)	—	Low	~2.0 (L)	3.5–5.0
Lactate (mmol/L)	—	3.5 (H)	—	0.5–2.0

Chest radiographs demonstrated bilateral basal atelectasis but no frank pneumonia. On day 17, sedation was continued, and a trial of feeding via the gastrostomy was initiated. The left upper quadrant drain was removed, and no new leakage was observed. A mild erythematous rash developed on both thighs, which was managed conservatively. On day 18, clear fluids via gastrostomy were initiated and tolerated. The pelvic drain was removed. The patient remained ventilated, sedated, and on TPN, with ongoing intensive care support.

## Discussion

Gastric perforation due to Candida albicans is exceedingly rare, with fewer than 15 cases documented in the literature to date. Reported cases typically occur in immunocompromised or critically ill patients, with diabetes, malnutrition, prolonged antibiotic use, and extended ICU stay being recognized risk factors [1]. Our patient had profound malnutrition and developed invasive candidiasis during a complicated ICU admission. Fungal peritonitis following gastrointestinal perforation carries high morbidity and mortality, with some studies citing mortality rates of 30-50% [1,2]. Several case reports and small cohorts have explored the diagnostic challenge, as bacterial peritonitis is more common and fungal cultures may take several days to yield results [4,5]. This diagnostic delay can lead to a lag in initiating antifungal therapy, which has been associated with worse outcomes [5]. In our case, Candida albicans was identified from intra-abdominal cultures within the first postoperative week. Despite early antifungal therapy, the patient deteriorated, necessitating re-exploration. The second operation highlighted the technical challenges of managing friable gastric tissue in the setting of ongoing sepsis. Placement of a gastrostomy tube through the site of perforation allowed controlled drainage and avoided a hazardous re-repair or a risky resection option. This pragmatic strategy, supported by prior case reports, can be lifesaving in situations where definitive repair or gastric resection is unsafe. This case emphasizes several important lessons. First, fungal etiologies should be considered in patients with refractory intra-abdominal sepsis after gastric perforation or even after any other intra-abdominal sepsis, particularly in those with recognized risk factors [1,4]. Second, timely initiation of antifungal therapy and individualized surgical source control are essential [2]. Finally, our experience aligns with prior reports in reinforcing the need for heightened clinical suspicion of fungal peritonitis in high-risk populations, as early recognition may alter the clinical course and improve survival [2-4].

In our search in multiple online databases for published cases of similar nature, only 28 total articles were found. Fifteen of them are case reports related to fungal infection leading to gastric ulcer and perforation, of which only six had confirmed Candida in body fluid analysis. Only four out of the six patients survived the post-op period, which further highlights the rarity of our case. The other 13 articles were guidelines, prospective cohorts, observational and narrative review studies.

The first case describes a middle-aged man who presented with gastric perforation where histopathology and culture confirmed Candida albicans. Despite aggressive management, the patient’s outcome was poor, illustrating the high mortality associated with invasive gastric candidiasis even in the absence of diabetes or immunosuppression [6]. Other authors have highlighted that non-albicans species can also cause similar pathology. A report of an immunocompetent adult with gastric perforation linked to Candida tropicalis, who improved after surgical repair and antifungal therapy, demonstrated that invasive gastric candidiasis is not confined to the classic immunocompromised host [7]. Several additional reports reinforce this variability. A description in which an elderly woman with posterior wall perforation - where yeast infiltration was seen throughout the gastric wall and C. albicans was confirmed from ascitic fluid - and distal gastrectomy with antifungal therapy achieved recovery [8]. In contrast, Prasad et al. documented an elderly man with C. albicans perforation who recovered following operative repair and fluconazole [9]. Pediatric involvement is rare, but Zhang reported an infant with gastric perforation due to C. albicans, successfully managed with surgery and antifungal treatment, highlighting the wide range of age and symptoms on presentation [10]. Beyond C. albicans, other fungi have been implicated. Termos et al. presented a patient with total gastric necrosis and perforation from mucormycosis requiring emergency gastrectomy and amphotericin therapy [11]. Similar cases from Sehmbey et al. [12] and Huang et al. [13] showed gastric ulcer perforation from mucor in both immunocompetent and adolescent patients. Aspergillus has also been described in Trésallet et al.'s study [14], which reported a large gastric perforation due to presumed primary digestive aspergillosis in an adult, while Reyna-Figueroa et al. [15] reported a pediatric case with gastric wall perforation during aplastic anemia therapy. Evidence from larger cohorts suggests that fungal involvement is not incidental. Lee et al. showed that nearly 40% of patients with perforated peptic ulcer peritonitis had Candida-positive cultures, with significantly worse outcomes than culture-negative patients [1]. Subsequent prospective studies confirmed that fungal co-infection is associated with higher morbidity and mortality in perforation peritonitis [4,5]. More recent multicenter analyses have questioned the routine use of empiric antifungals, noting no consistent mortality benefit once surgical source control is achieved [16,17]. Current guidelines, including those from the World Society of Emergency Surgery, therefore recommend selective antifungal use, reserving therapy for high-risk or culture-proven cases [3,18].

At the time of manuscript preparation, the patient remains intubated and under intensive care management, gradually stabilizing after the second laparotomy. Long-term follow-up and outpatient outcomes are not yet available. This represents a limitation of our report; however, the detailed description of the peri-operative challenges, intensive care course, and review of the existing literature still provides valuable insight into the management of this rare surgical entity.

Together, these reports show that fungal gastric perforation is an uncommon but increasingly recognized phenomenon, affecting both immunocompromised and immunocompetent hosts [1]. While C. albicans remains the predominant species, mucor and aspergillus can also cause full-thickness gastric necrosis with perforation [3]. Outcomes vary widely, but overall prognosis is guarded, particularly when compounded by systemic illness such as diabetic ketoacidosis or sepsis.

## Conclusions

Candida albicans gastric perforation remains an exceptionally rare surgical emergency, with very few cases described in the literature. Our case illustrates both the diagnostic challenge and the complexity of management when friable gastric tissue precludes definitive repair. Tube gastrostomy, combined with aggressive antifungal therapy and intensive supportive care, offered a practical surgical solution and allowed stabilization. This experience highlights the importance of maintaining clinical suspicion for fungal infection in refractory peritonitis and contributes to the limited evidence guiding surgical decision-making in such high-risk scenarios.
